# Training providers to improve access to emergency contraception in abortion ban states in the U.S.

**DOI:** 10.1186/s12978-026-02369-y

**Published:** 2026-05-27

**Authors:** Rachel Mundaden, Alejandra Alvarez, Yasaman Zia, Alison Comfort, Kristyn Brandi, Suzan Goodman, Cynthia C. Harper

**Affiliations:** 1 School of Medicine, University of California, San Francisco, San Francisco, CA USA; 2 Department of Obstetrics, Gynecology and Reproductive Sciences, University of California, San Francisco, San Francisco, CA USA; 3 San Francisco School of Medicine, Bixby Center for Global Reproductive Health, University of California, San Francisco, CA USA; 4 Luminosas Wellness Collective, Jersey City, NJ USA; 5 Department of Family and Community Medicine, University of California, San Francisco, San Francisco, CA USA

**Keywords:** Emergency contraception, Povider training, LNG-ECP, UPA, Copper IUD, LNG-IUD

## Abstract

**Background:**

Disparities in abortion and contraceptive care have intensified across the U.S. post-*Dobbs*, particularly in states with abortion bans and limited access to full-scope services. However, efforts to expand emergency contraception (EC) access and provider education have been limited. This study evaluated the impact of an evidence-based training on provider ability to offer the full range of EC methods.

**Methods:**

We developed and delivered a training intervention for providers, from 2022 to 2024, at 283 clinics in Texas, Oklahoma, and Indiana, all abortion restrictive states. Participants (*N* = 507) completed online surveys at baseline and three months post-intervention on clinical practices for EC levonorgestrel pills (LNG ECP), ulipristal acetate (UPA), copper intrauterine devices (IUDs), and levonorgestrel 52 mg IUDs (LNG IUD). We used generalized estimating equations to assess changes in provider attitudes, counseling, and availability of each type of EC.

**Results:**

Providers reporting that they strongly agreed that EC was safe increased across all methods, with the largest gains for UPA (58% to 78%; adjusted odds ratio [aOR 2.58], 95% confidence interval [CI] 1.37–4.89) copper IUDs (65% to 75%; aOR 1.76, 95% CI 1.03-3.00) and LNG IUDs (38% to 62%; aOR 2.71, 95% CI 1.91–3.83). More providers reported they included EC when counseling on contraception, with significant increases for IUDs for EC, from 61% to 77% for copper IUDs (aOR 2.39, 95% CI 1.38–4.13) and 44% to 64% for LNG IUDs (aOR 2.26, 95% CI 1.50–3.42). Counseling on pills was already high but increased further (LNG ECPs and UPA: 89% to 96%; aOR 2.78, 95% CI 1.87–4.15). Overall EC availability in clinic also improved, with LNG ECPs reaching universal availability (91% to 100%), and significant increases in UPA (67% to 79%; aOR 1.64, 95% CI 1.37–1.96) and IUDs (64% to 76%; aOR 1.77, 95% CI 1.07–2.91). Advance provision of EC pills increased modestly (28% to 34%; aOR: 1.40, 95% CI: 1.06–1.83).

**Conclusion:**

This replicable training intervention was associated with increased provider counseling and clinic availability of EC. Remaining gaps to reach universal provision of EC methods highlight the need for ongoing initiatives to increase access, including in abortion ban states.

## Background

Following the *Dobbs v Jackson Women’s Health Organization* decision, restrictive abortion policies have been enacted in many states, including some without exceptions for rape or incest [1, 2]. Limiting access to abortion can increase the urgency of contraceptive access for people wanting to prevent pregnancy, but recent research has shown that contraception has become even less accessible in abortion ban states [3, 4]. Within this context, emergency contraception (EC) – which includes both oral medications and intrauterine devices (IUDs) used after unprotected intercourse to prevent pregnancy – represents an important option to support reproductive autonomy [5].

EC is a unique advancement in reproductive healthcare that helps to prevent pregnancy following unprotected intercourse [6]. EC includes two types of oral pills, levonorgestrel (LNG ECP) and ulipristal acetate (UPA), as well as the copper intrauterine device (IUD) and the levonorgestrel 52 mg IUD (LNG IUD) [6, 7]. LNG ECP is available over-the-counter and can be taken up to 72 h or 3 days following unprotected intercourse [8]. UPA requires a prescription, can be taken up to 5 days afterward, and is the most effective pill regimen [9]. The copper IUD and LNG IUD can be placed up to 5 days after intercourse for EC purposes and can act as long-acting reversible contraception [7, 9]. IUDs as EC have no weight limits for effectiveness, unlike LNG ECP and UPA, which may become less effective as weight increases (8, 9, 10]. Increasing provider knowledge about the correct indications for all types of EC – particularly among providers in primary care, school-based health centers, and family planning clinics - can improve access to a range of options suitable for individual patient needs and facilitate strategies such as advance provision.

Primary care providers, school-based health centers, and family planning clinics play a critical role in ensuring timely access to EC, as they are often the first point of contact for patients seeking contraceptive care [11–14]. In addition to point-of care provision, these settings are well positioned to offer advance provision of EC – providing patients with EC in advance of need – which has been shown to increase timeliness of use. Strengthening provider capacity in these settings is therefore essential to improving access to EC in the current healthcare landscape [15, 16].

There is a notable knowledge gap among healthcare providers regarding the types of available EC and appropriate indications for use. A 2016 survey of emergency department (ED) providers in Georgia found that only 3% correctly identified the window of EC effectiveness, despite this being crucial treatment information [17]. Similarly, a 2016 study surveying reproductive health specialties reported that only 52% of providers had heard of UPA, and just 14% provided it to patients [18]. These findings suggest that gaps in knowledge may contribute to limited use of the most effective EC methods in clinical practice. Consistent with this, a 2020 national survey of OB/GYNs found that only 18% had ever prescribed UPA and only 29% had ever recommended a copper IUD as EC in their practice [19]. Together, this evidence indicates that improving provider knowledge may be an important step toward increasing appropriate counseling and access to EC, particularly given that, dissemination of innovations in medicine can be slow and falter without interventions to catalyze change [20, 21].

Provider counseling is an important source of trusted information for patients. The majority of available online EC resources have been shown to be low in credibility or reliability, and use language inaccessible for the average population seeking health information [22]. One study in 2021 showed that among adolescent and young adult females, 80% of participants had heard about emergency contraceptive pills (ECP); however, only 44% knew that ECP could be accessed over-the-counter, and only 28% knew it could be obtained without parental consent [23]. A study of 18–25 year olds visiting Planned Parenthood health centers showed that only 7.5% of them knew about IUDs as a form of EC [24]. However, a recent low-cost intervention showed young people can easily understand information about EC options and how to access them when given appropriate education [25].

EC is often mis-portrayed as an abortifacient, despite clinical evidence that LNG ECP acts by preventing ovulation and both LNG ECP and UPA at EC dose have no effect on implantation [26, 27]. The copper IUD and LNG IUD as EC prevent fertilization through their impact on sperm function; therefore, they also do not interfere with an already implanted embryo [7, 28]. Misinformation and confusion have only increased following the *Dobbs* decision, limiting EC provision and preventing people from accessing essential healthcare [29]. This widespread misinformation about EC has led to 11 state legislatures enacting laws to protect a pharmacist’s right to refuse to dispense medication if it violates their personal or religious beliefs [30]. To combat this misinformation and protect access to Plan B One-Step^®^, the brand name for LNG ECP, the Food and Drug Administration recently changed the package labeling to clarify that the drug mechanism only delays ovulation, does not disrupt an already implanted pregnancy, and therefore is not an abortifacient [31].

Following the *Dobbs* decision, birth control and EC prescriptions have significantly declined in abortion-restrictive states [32, 33]. Many family planning clinics providing abortion were forced to close soon after the *Dobbs* decision, often affecting contraceptive access as well [32]. Providers and patients alike have become fearful of future legal restrictions on contraception [4, 34]. The great majority of people (91%) report that a medical provider is their most trusted source of information about EC, thus demonstrating the high importance of investing in provider education [24]. This study evaluated the impact of an evidence-based provider training intervention about EC by examining changes in provider attitudes about the safety of different EC methods, counseling patients on EC, and availability of each method at their practice.

## Methods

We vdesigned, piloted, and delivered an evidence-based training for healthcare providers to increase their ability to offer people the full range of EC methods. The intervention is guided by the theory of dissemination of innovations in medicine, which emphasizes the need for active intervention to achieve clinical change [20]. EC is a primary example, as several methods have been approved for years, yet not all methods are universally available. In this context, we held 6 training sessions between 2022 and 2024. The sessions were delivered to a total of 712 providers practicing across 283 clinics in Texas, Oklahoma, and Indiana, all of which are abortion ban states. All sessions occurred after each state’s abortion ban was implemented. Texas and Oklahoma have no rape exceptions to their ban and Indiana has a limit for up to 10 weeks after “fertilization” [35]. There are over 8,550,000 reproductive-aged individuals assigned female at birth living in Texas, Oklahoma, and Indiana.

We established partnerships with health agencies in each state, and providers and clinic staff were invited to the voluntary training based on their health center’s participation in a health network or the referral network of our organizational partner. Healthcare providers who participated in the training included the full care team, with physicians, advanced practice clinicians, nurses, medical assistants, health educators, and social workers (all referred to as providers here). Practice settings for these providers included primary care facilities, youth and school-based health centers (SBHC), family planning clinics, and hospitals. The majority were Medicaid providers (61%), with 42% Title X recipients and 25% Federally Qualified Health Centers.

We collected data through online surveys before the training at baseline and 3 months after the training to measure the intervention’s impact on provider attitudes about safety, counseling on EC methods, and availability at their practice. For baseline data collection, emails were sent to individuals registered for the training one week prior to the training, with two reminder emails pre-training. For follow-up data, emails were sent to individuals who took the training at 3 months post-training, with two additional weekly reminder emails. Participants who delivered direct patient care or education were eligible for the survey, and the survey was designed with items that both clinicians and clinic staff could answer. Survey data to describe sample characteristics are also collected from participants, including provider type, provider race and ethnicity, practice setting, and location.

### Provider training intervention

We developed and piloted a training intervention for providers with the aim of expanding their clinical practices to include counseling and provision of a range of EC options. The intervention is an evidence-based curriculum covering all methods of EC; it was reviewed by national experts and a training advisory board, and accredited as a University of California, San Francisco School of Medicine Continuing Medical Education (CME) course. It is approximately one hour long and includes case studies, updated evidence, contraindications, and interactive opportunities to ask questions. The training intervention was held virtually to increase accessibility for providers across geographic areas. Participants completing surveys after the training session were entered into quarterly raffles for $250 gift cards.

The study protocol was approved by the University of California, San Francisco Institutional Review Board (UCSF IRB Protocol Number: 12-10336).

### EC outcome variables

We assessed provider attitudes on EC safety by type, including LNG ECP, UPA, the copper IUD, and LNG IUD. We asked participants whether they agreed or disagreed that each type of EC was safe, with responses on a 4-point Likert scale (strongly agree, agree, disagree, strongly disagree). In regression analyses, we measured changes in strongly agree compared to other responses. To assess EC counseling practices, questions were posed with a 4-point Likert scale to gauge how frequently counseling for each method of EC occurred when counseling people on contraception (never, sometimes, usually, always). In regression analysis, we modeled sometimes, usually, always compared to never (yes vs. no). To assess the availability of EC provision at the participating clinics, participants selected the various ways they provided each EC method, with responses including “provides onsite,” “gives advanced provision onsite,” “prescribes,” “gives advanced prescription,” “refers to pharmacy,” “gives referrals,” or none. While we provide frequencies pre and post for each category, in regression analyses, we combined responses on different modes of availability for sufficient power to compare overall whether it was available to people (yes vs. no). For many of the health centers in the sample including school-based health centers and primary care, improved counseling and referrals were hypothesized, especially in the case of IUDs. We also estimated any change in advance provision for emergency contraceptive pills, including prescriptions in advance for UPA (yes vs. no).

### Data analysis

Analyses include data from participants who delivered direct patient care or education. We presented descriptive statistics of the sample and of each of the EC outcome variables by method type. We assessed variation in our outcomes for EC safety, counseling, and method availability from pre- to 3 months post-training. We used generalized estimating equations for repeated measures to assess the impact of the intervention on each EC outcome, while controlling for provider type (clinician vs. non-clinician) and accounting for clustering by training. All analyses were conducted using Stata 16.0. Results were considered significant at the *p* ≤ 0.05 level.

## Results

### Sample characteristics

A total of 712 providers participated in the training, and of these participants, 596 were eligible because they delivered patient care or education. Response rate among the 596 eligible participants was 74% (442/596) at baseline, and 29% (142/442) at 3-month follow-up survey. About half of the sample were clinicians (52% physicians and advanced practice clinicians), and half were nurses, medical assistants, health educators, and social workers (Table 1). Of the sample, 57% identified as White, 13% as Hispanic/Latinx, 11% as African American/Black, 5% as Asian, and 14% as other.

**Table 1:** Characteristics of provider sample, pre and post intervention

Total	Pre	Post
	*N* = 442 (%)	*N* = 126 (%)
Provider Type	*N* = 442	*N* = 126
Clinician	232 (52%)	71 (56%)
Non-clinician	210 (48%)	55 (44%)
Practice Setting	*N* = 436	*N* = 119
Primary Care/ Health Department	129 (30%)	41 (34%)
Youth	158 (36%)	48 (40%)
Family Planning	104 (24%)	19 (16%)
Hospital/ Other	45 (10%)	11 (9%)
Race/ Ethnicity	*N* = 395	*N* = 115
African American/ Black	42 (10%)	11 (10%)
Asian	20 (5%)	4 (3%)
Hispanic/ Latinx	51 (13%)	13 (11%)
Middle Eastern	4 (1%)	0 (0%)
White	228 (58%)	68 (59%)
Multiracial/Other	24(6%)	14 (12%)
Prefer not to respond	26 (7%)	5 (4%)
Location	*N* = 421	*N* = 117
Urban	287 (68%)	78 (67%)
Suburb	75 (18%)	18 (15%)
Small town	39 (9%)	16 (14%)
Rural	20 (5%)	5 (4%)
State	*N* = 442	*N* = 126
Oklahoma	269 (61%)	64 (51%)
Indiana	93 (23%)	39 (32%)
Texas	80 (18%)	23 (18%)

Providers who participated in the intervention were from diverse clinic practice settings, with 30% practicing in a primary care setting, 36% in a youth or school-based health center, 24% in a family planning center, and 10% in a hospital or other setting. This intervention was offered to providers in abortion restrictive states, including Texas (18%), Oklahoma (61%), and Indiana (23%). Most participants reported their practices (68%) were in urban areas, 18% in suburban areas, and 14% in small town/rural areas (Table 1).

Providers reported that it was common for their patients to have concerns about EC as an abortifacient. Almost all participants (99%) reported their patients would benefit from easy-to-understand information on emergency contraception, and 84% thought there would be an increased need for patients to access emergency contraceptive pills.

An attrition analysis of provider characteristics showed relatively stable responses across categories for provider race and ethnicity and provider type. For practice setting, however, participants from family planning clinics were relatively less likely to respond to the post survey (family planning accounting for 24% of respondents in the pre vs. 16% in the post), and those from primary care (30% vs. 34%) and youth or school-based clinics (36% vs. 40%) were somewhat more likely to respond.

### Provider attitudes on safety

Most providers agreed that LNG ECP was a safe method, with the proportion of providers who strongly agreed increasing from 71% to 81% post-intervention (Table 2). Similarly, provider attitudes on the safety of UPA increased from 58% to 78%, of copper IUD from 65% to 75%, and of LNG IUD from 38% to 62%. Results from multivariable regression modeling controlling for provider type and clustering by training, showed a significant increase in providers strongly agreeing about the safety of the method for UPA (adjusted odds ratio (aOR): 2.58, 95% confidence interval (CI): 1.37–4.89), copper IUD (aOR: 1.76, 95% CI: 1.03-3.00), and LNG IUD (aOR: 2.71, 95% CI: 1.91–3.83) (Table 2).

**Table 2:** Emergency contraception: Perceptions of safety, Counseling frequency, and Availability by method type, Pre and post intervention

Outcome	Pre (%)	Post (%)	Adjusted OR
EC Safety^1^			
LNG ECP	71% (305/432)	81% (102/126)	1.81 [0.93–3.51]
UPA ECP	58% (249/429)	78% (98/126)	2.58** [1.37, 4.89]
Copper IUD	65% (280/433)	75% (94/125)	1.76* [1.03, 3.00]
LNG IUD	38% (165/429)	62% (77/125)	2.71*** [1.91, 3.83]
Counsels patients on EC^2^			
ECP	89% (373/418)	96% (119/124)	2.78*** [1.87, 4.15]
Copper IUD	61% (262/428)	77% (96/124)	2.39** [1.38, 4.13]
LNG IUD	44% (189/426)	64% (79/124)	2.26*** [1.50, 3.42]
Availability of EC Methods^3^			
LNG ECP	91% (400/442)	100% (126/126)	N/A^4^
UPA ECP	67% (294/442)	79% (99/126)	1.64*** [1.37, 1.96]
IUD as EC	64% (271/423)	76% (93/122)	1.77* [1.07, 2.91]
Advance provision EC pills	28% (124/442)	34% (43/126)	1.40* [1.06, 1.83]

^1^ Thinks EC method is safe: Strongly agree that EC is safe

^2^ Counsel on EC method: Yes (sometimes/usually/always) vs. No (never)

^3^ Available; Yes (provides onsite, prescribes, referral) vs. No; Advance provision (provides onsite in advance or UPA prescription in advance)

^4^ Regression analysis N/A due to universal availability, 100%, and therefore no variation on the outcome

*** *p* < 0.001, ** *p* < 0.01, * *p* < 0.05

### Counseling practices

The proportion of providers reporting that they discuss ECPs while counseling on birth control increased from 89% to 96% post-intervention. Similarly, the proportion of providers discussing the use of copper IUD as EC increased from 61% to 77%, and LNG IUD as EC from 44% to 64%. Multivariable models showed that, at 3 months following the training, there was a significant increase in providers who counselled people on ECPs (aOR: 2.78, 95% CI: 1.87–4.15), the copper IUD as EC (aOR: 2.39, 95% CI: 1.38–4.13), and the LNG IUD as EC (aOR: 2.26, 95% CI: 1.50–3.42) during appointments when discussing contraception and pregnancy prevention (Table 2).

### Provision practices

The proportion of participants reporting method availability at their clinic increased for all EC methods, including from 91% to 100% for LNG ECP, 67% to 79% for UPA, and 64% to 76% for both copper and LNG IUDs as EC post-intervention. Advance provision of EC pills changed from 28% to 34% at follow-up. When looking specifically at what kinds of changes the clinics made in terms of EC availability, onsite provision of LNG ECPs was stable (661% to 62%), with increases in prescriptions, which can be helpful for insurance coverage (37% to 47%), sending patients to pharmacy (27% to 38%). For UPA onsite provision increased (29% to 36%) as well as prescriptions (39% to 51%). For IUDs for EC, referrals was the category that increased the most from 19% to 30%, whereas only a few increased onsite 42% to 44%, as many clinics were school-based health centers. Multivariable regression results showed an increased odds of EC availability at the clinics 3 months post-training for UPA (aOR: 1.64, 95% CI: 1.37–1.96) and IUD as EC (aOR: 1.77, 95% CI: 1.07–2.91) (Table 2). For advance provision of EC pills, the increase was also significant (aOR: 1.40, 95% CI 1.06–1.83).

## Discussion

In the current legal landscape, where abortion access is limited in many US states, this study evaluates the feasibility of a provider-focused EC training and its association with provider-reported clinical practice changes within abortion ban states. Through educating healthcare providers about the range of EC methods and the evidence base surrounding these methods, this intervention was associated with increases in self-reported knowledge surrounding the safety of all EC methods, increased counseling about EC during contraception consultations, and reported increases in clinic-level availability of all EC options.

At baseline, provider awareness of the safety of EC methods beyond LNG ECP was limited. However, following this intervention, providers reported improved understanding of the safety of all EC methods, particularly UPA and IUDs, which are often underutilized in counseling. These changes were accompanied by increased self-reported inclusion of all EC methods in birth control counseling and increased perceived availability of UPA and IUDs, while LNG ECPs were reported to have become universally available at participating clinics. Including EC in contraceptive counseling is important even in primary care settings, so patients learn about their options for unprotected intercourse and in case of sexual assault. Research has estimated that 64,000 pregnancies resulted from rape in abortion restrictive states by 2024 since Dobbs [2]. These findings suggest that targeted educational interventions may help address knowledge gaps and support more comprehensive EC counseling, though outcomes are based on self-report from participating providers.

Despite these reported improvements, gaps remain. Providers indicated that IUDs are still less frequently discussed as EC compared to oral methods. While IUDs are the most effective type of EC, ECPs are more commonly used, likely due to factors such as individual patient preference, familiarity, ease of access, limits in public knowledge, and advice from peers [34]. These findings highlight ongoing barriers to translating knowledge gains into consistent clinical practice, which should be further explored.

Differences across EC methods further illustrate important knowledge gaps. At baseline LNG ECP was the most well-known and trusted method of EC among providers, and the most frequently reported option in counseling, as expected with previous studies [35]. However, LNG ECP is limited by a 72-hour window of effectiveness and reduced efficacy for people above certain weight thresholds. In contrast, fewer providers reported awareness of UPA compared to LNG ECP at baseline, a gap seen in prior research but not yet addressed with interventions [35]. This is a noteworthy knowledge gap, considering UPA is the most effective pill regimen, with a longer window of use and a higher weight limit [35–37]. However, following this intervention, providers reported increased counseling and availability of UPA, suggesting improved awareness of its role as an alternative oral EC option.

Similarly, knowledge on safety of IUDs for EC was lower at baseline, despite strong evidence of effectiveness, regardless of body size. Perceptions of safety around IUD for EC was also lower than those for LNG ECP and UPA at follow-up, despite increases across the board. In addition, while reported increases in advanced provision were observed, adoption of this practice remained limited, indicating potential barriers to full integration of this practice. These findings may indicate the utility of follow-up sessions to troubleshoot challenges that arise as clinics move to change practices.

Following our intervention, providers reported increased preparedness to counsel patients on this option. Previous research has demonstrated interest in same-day IUD placement for EC among people who receive education about this option [38]. While same-day placement may not always be feasible, counseling during routine contraceptive visits may allow patients more time to consider options before urgent need arises, potentially supporting more informed decision making.

Beyond counseling and method-specific knowledge, changes in prescribing and provision practices were observed. This study highlights the potential of advanced provision of ECPs – defined as providing a supply or prescription in advance of need – to improve the timeliness of EC use [39, 40]. Providers reported increased use of advanced provision strategies following the intervention, suggesting a shift in clinician-reported practices. This may represent an early indicator of changes in clinician-reported practices relevant to timely access of EC, though patient-level outcomes were not measured.

Structural and systems-level barriers may also influence implementation of this intervention. While providers reported universal availability of LNG ECP at follow-up, availability of UPA and IUDs as EC remained substantially lower. Given that availability in this study included onsite provision, prescriptions, and referrals, this disparity may reflect differences in cost, insurance coverage, staffing capacity, and clinic infrastructure. In particular, IUD for EC depends on trained staff and the capacity for same-day insertion, and our follow-up period may not be long enough to witness these changes. Patient-level factors, including limited awareness or comfort with different EC methods, may also contribute to barriers to uptake. Future research should further examine structural barriers and patient-level barriers to better understand how to expand access to the full range of EC options.

These findings must also be interpreted within the broader post-*Dobbs* landscape. Following the *Dobbs* decision, many states have enacted laws that restrict abortion care, contributing to increased reproductive health risks, including delays in care for ectopic pregnancies, miscarriages, nonviable pregnancies, and other complications of pregnancy [41]. In this context, providers may face additional legal and institutional constraints that affect timeline delivery of reproductive health services [42]. This study suggests that targeted educational interventions may represent one potential strategy for supporting provider capacity in such restricted settings, particularly for time-sensitive services such as EC.

This study has limitations. A primary limitation is that the response rate for the post-intervention surveys was lower than expected, introducing potential attrition bias. In particular, providers from family planning clinics were less likely to respond to the follow-up survey. As family planning providers may have higher familiarity with EC, and therefore less room for change, their underrepresentation at follow-up could have accentuated the observed improvements in provider knowledge, counseling, and reported EC availability. While the ultimate access goal is to have EC available onsite, for this study we measured improvements that also included prescriptions and referrals, as many sites were primary care and school-based health centers, rather than specialist reproductive health centers, which have often been de-funded in abortion ban states. It is also important to note that the generalizability of this intervention may be limited since participants were self-selected to participate in this voluntary training, which likely reflects a more motivated provider population than the broader workforce in abortion ban states. Furthermore, a 12-month follow-up would lend further insight into long-term knowledge uptake and clinical practice change in these settings. Finally, the measure of method availability was inclusive of on-site, prescriptions, and referrals, and based on provider reports, therefore it may not capture actual patient-level access to EC methods.

In summary, this provider-focused EC training was associated with improvements in self-reported counseling practices, knowledge of EC methods, and reported availability of various EC options. While these findings suggest potential for educational interventions to support provider practice change, they also highlight persistent gaps in implementation and access. Further research using longer follow-up periods, objective measures of provision, and patient-level outcomes is needed to better understand the impact of such interventions on reproductive healthcare access in settings where such access has become more limited.

## Conclusion

Few interventions have addressed EC provision in settings with restrictions to reproductive health care. In this study, a provider-focused educational training was associated with increases in self-reported knowledge of EC method safety, EC counseling practices, and reported availability of EC methods, including improved access to UPA and IUD-based options alongside widespread availability of LNG ECPs. While these findings suggest that targeted trainings may support provider practice change in restrictive settings, persistent gaps in reported knowledge, counseling practices, and availability across methods highlight the need for additional strategies to support equitable access. Further research is needed to assess long-term effects and patient-level outcomes.

## Data Availability

The data generated and analyzed for this study will be deposited in a restricted repository upon acceptance of this manuscript for publication. The data that support the findings of this study are available from the corresponding author upon reasonable request.
